# Comparative assessment of two participatory approach methods for selecting pig health indicators: A proof-of-concept on respiratory health

**DOI:** 10.1371/journal.pone.0358467

**Published:** 2026-09-17

**Authors:** Théo Dessier, Nikky Millar, Emélie Suazo, Céline Tallet, Catherine Belloc, Mily Leblanc-Maridor

**Affiliations:** 1 Oniris INRAE, BIOEPAR, Nantes, France; 2 PEGASE, INRAE, Institut Agro Rennes Angers, Rennes, France; University of Life Sciences in Lublin, POLAND

## Abstract

Over recent years, antimicrobial use (AMU) in pig farming has substantially declined across Europe, including France, following regulatory measures and sector-wide initiatives. Maintaining these reductions while preserving pig health and welfare remains a major challenge. Stakeholders have therefore highlighted the need for a structured on-farm health monitoring protocol to support judicious AMU. However, no comprehensive set of pig health indicators has yet been systematically assessed and selected for this purpose. Expert elicitation offers a promising approach to address this gap. This study aimed to develop and compare two participatory methods for mobilizing expert knowledge to identify and characterize relevant pig health indicators while making efficient use of experts’ time. Both methods followed the same six-step framework but differed in their implementation. In each method, experts assessed indicators according to four attributes (feasibility, reliability, specificity, and sensitivity) and subsequently discussed the results to reach agreement on a final set of relevant indicators. Porcine respiratory health was used as a proof-of-concept to compare the performance and outputs of the two methods and select the most appropriate one. Both methods enabled experts to reach agreement and generated well-characterized indicators, supported by experts’ rationales. Although Method B required greater expert involvement, it produced a more comprehensive set of 52 relevant respiratory health indicators and allowed greater flexibility in how experts allocated their time throughout the process. This participatory approach method provides a practical framework that encourages expert involvement and allows the selection and characterization of relevant pig health indicators. The method will be extended to other pig health systems beyond respiratory health.

## Introduction

Over the past decades, growing awareness of antimicrobial resistance (AMR) has driven substantial reductions in antimicrobial use (AMU) in animal health [1–3]. However, decreasing AMU without implementing adequate preventive measures may compromise animal health and welfare [4].

In 2022, the ROADMAP project funded by the European Union’s Horizon 2020 explored approaches to promote a responsible AMU while preserving animal health and welfare. As part of this project, a Living Lab brought together researchers, veterinarians, representatives of the pig and the poultry production chains and public authorities to co-design practical solutions for the pig and the poultry sectors [5]. The exchanges resulted in a shift in perspective. Rather than focusing solely on reducing AMU, participants emphasized on the importance of achieving an optimal AMU, *i.e.,* a prudent and context-specific use that preserves animal health and welfare [5,6]. To support this transition, stakeholders underlined the need for a structured protocol capable of jointly assessing AMU, animal health and welfare as well as their interactions and dynamics over time. Such a protocol would support the identification of health issues and guide preventive management decisions.

However, stakeholders involved in the Living Lab highlighted practical difficulties in developing such protocols, particularly due to the need to identify consensual indicators capable of capturing on-farm health situations [5]. Although numerous scientifically valid indicators (*i.e.*, indicators that accurately measure the phenomenon of interest) are proposed in the literature, only a small proportion has been actually evaluated under field conditions [7,8]. Health indicators that perform well under experimental conditions may not be suitable for routine on-farm use, where health status is influenced by complex interactions among pathogens, management practices, and environmental factors [9]. Beyond scientific validity, indicators must therefore also be relevant for field implication. In the scientific literature relevance refers to the extent to which an indicator is useful, applicable, and aligned with stakeholders’ needs in real-world conditions [8,10]. Accordingly, in this study, an indicator is considered relevant if, beyond being scientifically valid, it demonstrates key practical attributes, including feasibility, reliability, specificity, and sensitivity under on-farm conditions [11,12].

Several studies regarding animal welfare have used expert elicitation to identify indicators, but none have addressed the selection of pig health indicators [13–15]. To address this gap, and following recommendations from the Living Lab, two participatory approach methods were therefore developed with swine veterinarians (*i.e.*, animal health experts and future users) to identify, assess and select relevant pig health indicators through the scoring of four attributes: feasibility, reliability, specificity and sensitivity [12]. Participatory approaches provide an effective way to identify relevant indicators when empirical evidence is limited [16–19]. By structured involvement of experts with domain-specific knowledge, these approaches ensure the identification of contextually appropriate indicators and increase the legitimacy and stakeholders’ approval of the results [16,20,21]. Nevertheless, a major methodological challenge is to use a method that effectively mobilizes veterinarian knowledge to select relevant health indicators while optimizing the limited time they can dedicate to such initiatives.

Therefore, the objective of this study was to comparatively assess two distinct participatory approach methods (Methods A and B) to determine which performs best by addressing these constraints. While both methods share the same six steps framework, their execution differs mainly in two aspects: the quantity of work requested from experts as well as how experts allocated their time during the approach and were guided. To evaluate the performance and results of both methods, the selection of pig respiratory health was used as a proof of concept. Respiratory disorders are one of the most prevalent and multifactorial health challenges in pig production and involve a wide diversity of potential indicators [22,23]. This paper demonstrates how expert knowledge can be efficiently used in a limited timeframe to lay the groundwork for subsequent field-measurement validations.

## Materials and methods

### Participatory approach methods’ design (step 1)

#### Conceptualisation of the two methods.

The developed participatory approach methods were inspired by established participatory frameworks, namely the Delphi method, Focus group, and the Nominal Group Technique [24,25].

**Fig 1:**
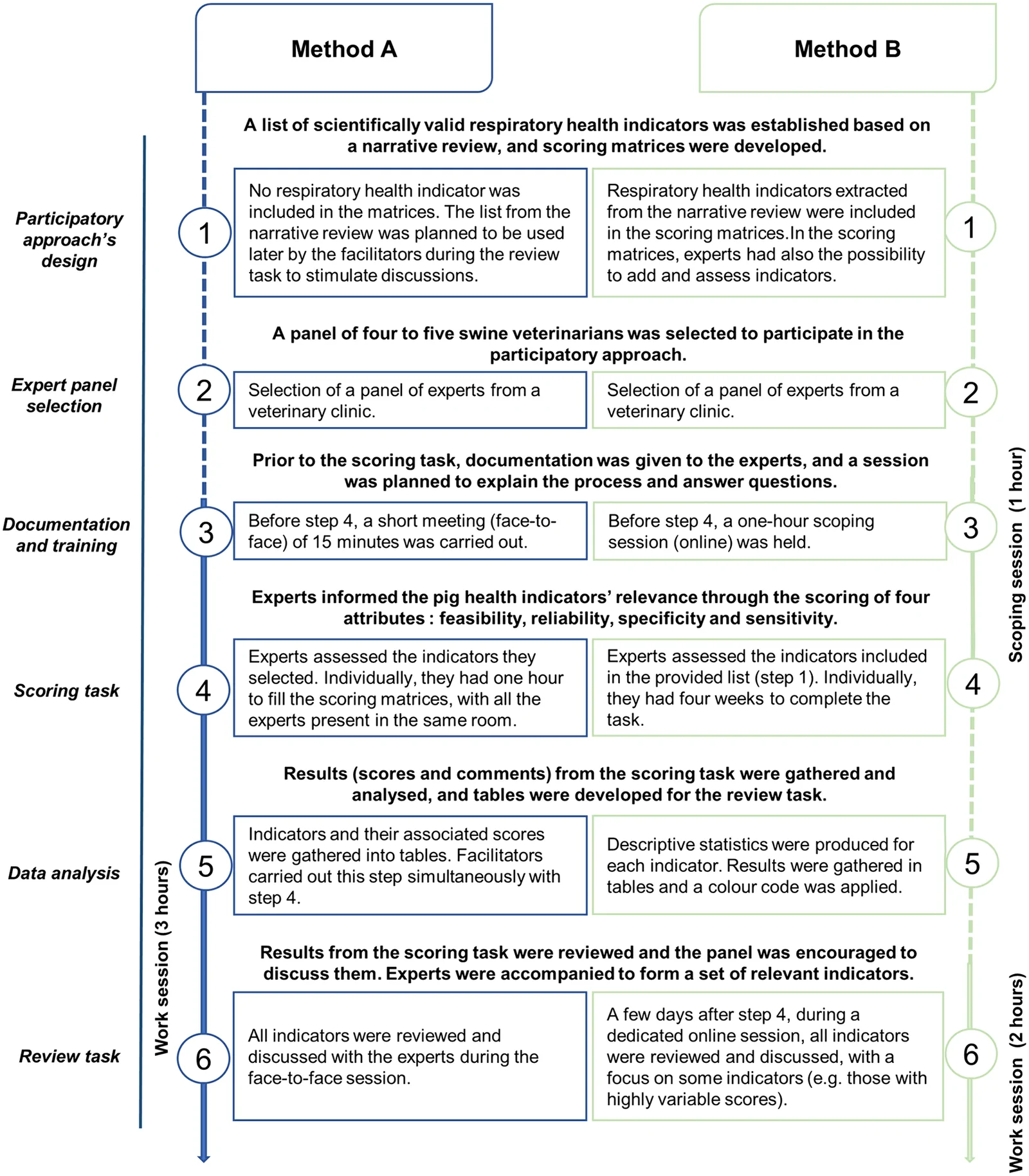
Comparative workflow of the two participatory approach methods (A and B) used for the assessment and selection of respiratory pig heath indicators by veterinarians.

Both methods shared a common six-step framework (Fig 1) and were managed by four facilitators specialized in veterinary medicine and animal welfare, two of whom were trained in participatory approaches and focus groups. In Method A, four steps (training, scoring task, data analysis, and review task) were condensed into a single 3-hour in-person session. During the scoring task, experts had the opportunity to freely propose respiratory health indicators and assess them. In contrast, Method B relied on a pre-defined list of indicators and was split into a 1-hour online scoping session (training), an asynchronous individual work (scoring task and data analysis), and a final 2-hour online session (review task).

#### Narrative review.

A comprehensive narrative review was first conducted in march 2024 across the PubMed and CAB Abstracts databases to identify scientifically valid pig health indicators. The search equation was: (« Indicators » OR « criteria » OR « assessment ») AND « health » AND (« swine » OR « pigs » OR « pig breeding » OR « pig farms » OR « pork »). Articles were selected if they evaluated pig health using one or several validated indicators. In total, 129 articles were reviewed. In addition, references coming from other sources were consulted to complete the list, such as Zimmerman’s reference book “Diseases of swine” [23]. The extracted indicators were firstly categorized by biological system (*i.e.*, respiratory, digestive, locomotor, urinary, nervous, reproductive or cutaneous). Within each system, indicators were further classified into six categories (clinical signs, biological analysis, slaughterhouse data, necropsy data, performance data and environmental data), encompassing both animal-based measures (ABMs; *i.e.*, indicators assessing animals’ reactions to their environment) and resource-based measures (RBMs; *i.e.*, indicators evaluating environmental factors and management practices). Table 1 presents the pre-defined list of pig health indicators obtained for the respiratory health system.

**Table 1:** List of respiratory health indicators in pigs extracted from the narrative review.

Clinical signs	Biological analysis	Slaughterhouse data	Necropsy data	Performance data	Environmental data
Abnormal vocalizations	Bacterial culture	Atrophy nasal turbinates	Atrophy nasal turbinates	Antimicrobial use	Ambient temperature
Anemia	Bacterial identification	Cardiac lesion	Cardiac lesion	Daily weight gain	Ammoniac concentration in the air
Bradycardia	Cell study	Hepatic lesion	Hepatic lesion	Feed conversion ratio	Health status of animals
Bradypnea	Complement fixation	Lymphatic node lesion	Lymphatic node lesion	Prevalence of morbidity	Batch mixing
Conjunctivitis	Complete blood count	Pleuritis	Pleuritis	Prevalence of mortality	Cleaning protocol
Coughing	Direct ELISA	Pneumonia	Pneumonia	Weight	Clothes types
Cyanosis	Genotyping	Pulmonary abscess	Pulmonary abscess		Carbon dioxide concentration in the air
Dehydration	Hemagglutination inhibition	Rhinitis	Rhinitis		Density
Dyspnea	Immunohistochemistry				Hygiene lock
Epistaxis	Immunofluorescence				Dust concentration in the air
Feeding difficulties	Indirect ELISA				Floor type
Growth retardation	In situ hybridization				Gilt quarantine
Huddling	Lung histology				Humidity
Hyperthermia	Lymphatic node histology				Hygiene of animals
Hypothermia	Mass spectrometry				Hygiene of the environment
Lethargy	Measure of anemia				Nearby pig farms
Mortality	Measure of blood oxygen				Quarantine of sick animals
Nasal discharge	Measure of blood parameters				Room disinfection
Orthopnea	Parasitological analysis				Room drying
Pain posture	PCR				Vaccination protocol
Panting	Viral isolation				Ventilation
Polypnea	Virus neutralization assay				
Rough hair coat					
Shivering					
Sneezing					
Tachycardia					
Tachypnea					
Twisted snout					

#### Development of the scoring matrices.

Scoring matrices were designed using Microsoft Excel (v1808). Their objective was to collect experts’ assessments of respiratory health indicators. A total of six scoring matrices were developed, each corresponding to a specific indicator category (clinical signs, biological analysis, slaughterhouse data, necropsy data, performance data and environmental data). The overall structure of the matrices was identical for both methods, with each row representing an indicator and each column an information to be collected. In Method A, the rows were intentionally left blank, allowing experts to propose and assess the indicators they considered relevant. In Method B, the rows were pre-filled with the respiratory health indicators identified through the narrative review. For each indicator, experts followed the same steps:

- They first provided characteristics on the indicators, i.e., whether they used it to investigate respiratory diseases or not. If not, they provided a justification and proceeded to the next indicator.
- If the indicator was used, expert specified the measurement method and the physiological stages to which it applied (suckling piglets, growing pigs, finishers, or sows).
- Experts, then, assessed the indicator’s relevance by scoring four attributes (feasibility, reliability, specificity, and sensitivity). Each attribute was scored through a statement adapted from definitions reported in the literature [12] (Table 2). Experts expressed their level of agreement with each statement by using a 4-point Likert scale ranging from 1 (strongly disagree) to 4 (strongly agree). To capture the multidimensional nature of respiratory health, specificity and sensitivity were scored in relation to common respiratory diseases.
- Experts were allowed to add any comments regarding their scores. In Method B, experts could also add and assess indicators that were missing from the pre-defined list.

**Table 2:** Definitions for the four attributes defining the relevance of pig respiratory health indicators and given to the veterinarians.

Attribute	Definition
Feasibility	Refers to the practicality of the indicator in terms of time, equipment, operators’ skills, and costs.
Reliability	Refers to the capacity of the indicator to provide consistent results, regardless of the operator, the equipment used, or the time of measurement.
Specificity	Refers to the capacity of the indicator to measure what is intended to be measured, and not something else
Sensitivity	Refers to the ability of the indicator to detect variations in health status, even when these variations are small

Tables 3 and 4 represent a scoring matrix provided to experts during the scoring task and adapted and formatted for publication purposes.

**Table 3:** Scoring matrix (indicator’s characteristics section) used by the experts during the scoring task.

Indicator	Use?	If not, why?	If yes, how?	Physiological stage			
				Suckling piglets	Growing pigs	Finishers	Sows
Coughing							
Sneezing							
Nasal discharge							
Other indicators							

Experts gave information regarding indicators’ characteristics (use and concerned physiological stage). The matrix was identical in both Method A and B. The last row “Other indicators”, where experts has the possibility to add and assess indicators that were not present, was only available for Method B.

**Table 4:** Scoring matrix (indicator scoring section) used by the experts during the scoring task.

Indicator	Feasibility	Reliability	Specificity						Sensitivity					
			Enzootic pneumonia	APP infection^a^	Influenza	PRRS^b^	Glässer disease	Atrophic rhinitis	Enzootic pneumonia	APP infection^a^	Influenza	PRRS^b^	Glässer disease	Atrophic rhinitis
Coughing														
Sneezing														
Nasal discharge														
Other indicators														

Experts scored indicators regarding their perceived feasibility, reliability, specificity and sensitivity for common respiratory diseases.

a Infection caused by *Actinobacillus pleuropneumoniae*.

^b^Porcine Reproductive and Respiratory Syndrome.

### Expert panel selection (step 2)

Experts were volunteer swine veterinarians, with varying field experience, all working at the same veterinary clinic. For each participant, years of professional experience and gender were recorded. All experts received financial compensation to acknowledge the workload associated with their participation. The same recruitment strategy was applied in both Method A and B: a motivated swine veterinarian was identified through the facilitators’ professional network and invited to participate. The veterinarian was, then, asked to recruit three to four motivated colleagues.

### Documentation and training (step 3)

Documentation (identical for both methods), written in French and describing the participatory approach, was sent to the experts by e-mail before the scoping session. This documentation aimed to familiarize participants with the project’s objectives, the participatory approach, the definitions of the four attributes defining the relevance of an indicator and the scoring matrices (S1 File). The overall documentation was designed to require approximately 30 minutes to review.

To minimize ambiguities before the scoring task, facilitators organized a dedicated scoping session for each method.

In Method A, a 15 minutes in-person meeting was conducted immediately before the scoring task.

In Method B, a 60-minutes videoconference scoping session was held one week after the documentation had been distributed. During this session, the facilitators presented the participatory approach and provided a live demonstration of how to fill the scoring matrices. Facilitators ensured that instructions were clearly understood before the scoring task and confirmed that experts remained engaged in the study.

### Scoring task (step 4)

The scoring task was implemented differently in the two methods.

In Method A it was conducted with all experts and facilitators present in the same room. Starting from blank matrices, experts had one hour to individually propose and assess respiratory health indicators. The filled matrices were collected and processed in real-time by the facilitators.

In Method B, experts received pre-defined scoring matrices containing the respiratory health indicators. They were given approximately four weeks to complete the assessment individually, after which the filled matrices were returned and processed by the facilitators. Throughout the entire approach, facilitators remained available to answer questions, and reminders were regularly sent to the experts.

### Data analysis (step 5)

For both methods, expert scores and qualitative comments were gathered into six matrices corresponding to the six indicator categories. No further data analysis was conducted for Method A. For Method B, experts scores were further summarized using descriptive statistics in Rstudio (v2024.12.0). For each indicator, the following parameters were calculated: (i) the percentage of experts using the indicator, reported for each physiological stage and (ii) the mean score and its standard deviation (SD) for each attribute.

To facilitate expert interpretation, a color code was applied to distinguish four categories of indicators: (i) low-score indicators (mean ≤ 2), (ii) mid-score indicators (2 < mean < 2.5), (iii) high-score indicators with low variability (mean ≥ 2.5 and SD ≤ 1), and (iv) high-score indicators with high variability (mean ≥ 2.5 and SD > 1).

### Review task (step 6)

The review task aimed to reach an agreement among the panel of experts on a set of relevant respiratory health indicators. One indicator at a time, experts discussed their agreement with the results (*i.e.,* scores and the associated comments). They were also encouraged to provide qualitative feedback to clarify their scoring and perception of each indicator.

In Method A, the review task was held immediately after the scoring task and each assessed indicator was discussed.

In Method B, the review task was carried out during an online session a few days after the end of the scoring task. To handle the large quantity of data, experts were first asked to briefly discuss (i) the indicators used by only one or two experts or with the lowest average ratings in order to exclude them from the selection, and (ii) the indicators used by the majority of experts with the highest average ratings in order to confirm their relevance. Discussions then focused on indicators with highly variable scores among the panel and those added by the experts.

For both methods, an indicator was included in the final set only if all experts unanimously agreed on its relevance. Both sessions were concluded on a final validation of the set by the entire panel.

### Ethics statement

This study complies with the ethics requirements imposed by the French National Research Institute for Agriculture, Food and Environment (INRAE) and by the French ministry of Agriculture. All participants were fully informed about the project’s nature, scope, and objectives, and their participation was strictly voluntary. Oral consent on the use of data was obtained during the panel selection, followed by formal written consent prior to the scoring task. For both methods, expert panel selection and data collection were conducted during the summer months of 2024. All data and names were anonymised.

## Results

Following the completion of the two participatory approach methods, their performance was comparatively assessed across two dimensions: the implementation of the method (*i.e.*, group dynamic, expert understanding, time management and flexibility) and the results of the respiratory health indicator selection (*i.e.*, number and characteristics of the selected indicators).

### Implementation of the participatory methods

#### Group dynamic.

Method A involved four veterinarians (two men, two women), whereas Method B involved five (three men, two women). Both panels included experts with different levels of professional experience (ranging from 4 to 39 years in Method A and from 5 to 30 years in Method B).

Regarding group dynamic, several points can be highlighted. Although experts in Method A were instructed to fill the scoring matrices individually, they interacted with each other and the facilitators numerous times. Nevertheless, all participants submitted their filled matrices within the allocated one hour period. During the review task, each indicator was discussed by the panel, although discussions were brief. Experts provided rationales and clarifications for their scores during these discussions**.** The pre-defined list of indicators, initially planned as a proxy to identify potentially missing indicators, was not used because the review task exceeded the allocated time. By the end of the task, the group reached agreement on a final set of respiratory health indicators.

During the online review task in Method B, experts discussed the results and provided rationales for their assessments. Differences in experts’ opinions regarding the scores of some indicators were identified and discussed by the panel. Agreement on whether indicators should be selected or excluded was reached during the discussion. By the end of the task, participants reached agreement on a final set of respiratory health indicators.

#### Expert understanding.

Experts suggested minor revisions to the documentation. In Method A, during the meeting before the scoring task, experts asked questions about the project and the documentation and requested additional information. At the end of the meeting, all experts confirmed that they understood the instructions.

In Method B, experts gave a positive feedback on the scoping session. They had questions mostly about the project or the documentation, and asked for more details on the aim of each step. One expert initially expressed concerns about the workload, but facilitators explained that the four-week scoring period allowed participants to complete the task at their own pace. Then, during the scoring task, two experts (out of five) contacted the facilitators for clarifications on the scoring matrices. Following the review task, experts also provided positive feedbacks regarding the results matrices and the colour coding system, which they considered useful for rapidly identify indicators of interest and those requiring discussion during the review task.

#### Time management.

In Method A, during the scoping session, answering experts’ questions (*e.g.*, regarding the project’s objectives and instructions) and confirming their willingness to participate required nearly 30 minutes instead of the 15 minutes initially planned. Despite this delay, the one hour scoring task was maintained as scheduled. However, two of the four experts reported that one hour was insufficient to fill and double-check the scoring matrices. They suggested allowing several days to carry out the task in subsequent sessions. One expert considered the possibility of adding comments to the scores particularly valuable, although time-consuming. Finally, as delays accumulated, less than an hour remained for the review task. Because each expert had proposed a different set of indicators during the scoring task, facilitators first had to collect feedback from the other experts before discussing each indicator. Consequently, experts could only briefly review the results, limiting opportunities for in-depth discussion and consensus building. Overall, experts from method A devoted approximately 3 hours to the participatory approach.

For Method B, the one-hour scoping session was sufficient to answer experts’ questions and demonstrate how to fill the scoring matrices. Moreover, following the scoring task, all scoring matrices were returned on time. All five experts reported that four-week completion period allowed them to organize their time and workload according to their own professional constraints. This period also allowed facilitators to carry out a detailed data analysis for the review task. Experts estimated the time to fill the matrices to be approximatly four hours. Regarding the online review task, all of the participants completed it within the two hours allocated. Overall, experts devoted approximately 7 hours to the participatory approach.

#### Flexibility.

Experts’ feedback during Method A showed that carrying out the entire participatory approach during a unique 3-hours meeting was not ideal. Moreover, regarding the scoring task, one expert reported difficulty identifying which indicators to include in the matrices. Ultimately, the whole panel indicated that pre-defined list of indicators should be preferred for this work, as it could save time during the scoring process and because it better reflects their routine approach to assessing pig health on farms.

In Method B, experts gave positive feedbacks regarding the division of the participatory approach into several sessions, especially the four-weeks scoring task, which was perceived as a strength. They also expressed no concerns about using a pre-defined list of indicators.

In addition, finding between experts and facilitators a common time for starting the participatory approach and perform the scoping session was easier and faster in Method B than in Method A.

### Selection of respiratory health indicators

The narrative review identified 93 respiratory health indicators, including 71 animal-based and 22 environment-based indicators. They covered all six predefined categories (clinical signs, biological analysis, slaughterhouse data, necropsy data, performance data and environmental data).

Following the scoring and review tasks, experts from both methods actively contributed by provinding information on indicators and their measurement methods. They identified a final set of respiratory health indicators considered relevant by the experts. The changes in the number of indicators in both methods is presented in Table 5.

**Table 5:** Evolution in the number of respiratory health indicators over both methods.

	Method A		Method B			
	Suggested and assessed	Selected	Initial	Assessed	Added and assessed	Selected
Clinical signs	10	10	28	26	2	14
Biological analysis	5	5	22	13	1	10
Slaughterhouse data	4	4	8	8	2	4
Necropsy data	4	4	8	8	2	6
Performance data	5	5	6	6	0	5
Environmental data	4	4	21	21	0	13
Total	32	32	93	82	7	52

In method A, during the scoring task, experts collectively proposed and assessed 32 indicators covering all six predefined categories (an identical indicator proposed by several was counted once). The number of indicators proposed by a given expert spanned from 13 to 26. All of them were present in the list of indicators derived from the narrative review. At the end of the review task, despite the brief discussions on each result, experts agreed on the relevance of the 32 assessed indicators (Table 6). Both on-farm clinical observations (*e.g.*, abnormal behaviours or biological analysis) and post-mortem measures (*e.g.*, organ lesions) were included in the set of indicators. Furthermore, the indicator set spanned both herd (*e.g.,* “mortality”, “total condemnations”) and individual-level of measurement (*e.g.,* “lethargy”). Finally, only four environmental indicators, related to the room ambiance, were proposed by the experts.

**Table 6:** Selected indicators in each category following the review task in both participatory methods.

Clinical signs	
**Method A**	Abortion; Breathing difficulties; Coughing; Epistaxis; Feeding disorder; Hyperthermia; Infertility; Mortality; Sneezing; Twisted snout.
**Method B**	Breathing difficulties; *Cool-seeking behaviour*; Coughing; Epistaxis; **Feeding disorder**, **Growth retardation**; Hyperthermia; **Lethargy**; Mortality; Nasal discharge; **Rough hair coat**; Sneezing; Twisted snout; ***Wasting away***.
**Biological analysis**	
**Method A**	Bacterial culture; Direct ELISA; Hemagglutination inhibition; Lung histology; PCR.
**Method B**	Bacterial culture; Bacterial identification; Direct ELISA; Genotyping; Hemagglutination inhibition; Indirect ELISA; Lung histology; Mass spectrometry; *Nasal cavity histology*; PCR.
**Slaughterhouse data**	
**Method A**	Deviated nasal septum; Pleuritis; Pneumonia; Total condemnations.
**Method B**	Nose scoring; Pleuritis; Pneumonia; ***Total condemnations***.
**Necropsy data**	
**Method A**	Cardiac lesion; Lymphatic node lesion; Pleurisy; Pneumonia.
**Method B**	Deviated nasal septum; Lymphatic node lesion; Pleurisy; Pneumonia; ***Pulmonary oedema***; Rhinitis.
**Performance data**	
**Method A**	Antimicrobial use; Daily weight gain; Feed conversion ratio; Herd heterogeneity; Prevalence of mortality.
**Method B**	Antimicrobial use; **Daily weight gain**; **Feed conversion ratio**; **Prevalence of mortality**; Weight.
**Environmental data**	
**Method A**	Ambient temperature; Ammoniac smell; Humidity; Ventilation.
**Method B**	Ambient temperature; Batch mixing; Cleaning protocol; Clothes types; Density; Dust concentration in the air; Floor type; Gilt quarantine; Health status of animals; Hygiene lock; Nearby pig farms; Quarantine of sick animals; Vaccination protocol.

Bold indicators were perceived as related to systemic health while italic indicators were added by experts during the scoring task.

In Method B, of the 93 indicators identified through the narrative review, 82 were reported as being used and were subsequently assessed. Two experts were however unable to fill the environmental data matrix because of personal circumstances. During the scoring task, four experts proposed seven additional indicators: “wasting away”, “cool-seeking behaviours”; “pulmonary oedema”, “tracheitis”, “deviated nasal septum”, “total condemnations” and “nasal cavity histology”. Consequently, a total of 89 indicators were assessed during the scoring task (S2 File). Following the review task, experts selected 52 indicators as relevant, including 47 from the initial list and 5 newly proposed (Table 6). During the discussion, they agreed to merge “dyspnea”, “orthopnea” and “polypnea” into a single indicator: “breathing difficulties”. Similarly, the slaughterhouse indicators, “deviated nasal septum” and “atrophy nasal turbinates” were combined into “nose scoring”. As in Method A, the final set included indicators collected on animals (*e.g.*, respiratory clinical signs and performance data) as well as indicators obtained from necropsy and slaughterhouse, at both individual and herd levels. Environmental data represented an important part (40%) of the final set of selected indicators. Finally, experts agreed that among these 52 indicators, 10 (including 3 added indicators) were not specifically related to respiratory health and should be therefore considered as relevant to assess systemic health in pigs.

Finally, in both methods, experts tended to score the specificity and sensitivity of an indicator for the most common diseases only. Although experts were initially instructed to score specificity and sensitivity for all diseases included in the scoring matrices, fill matrix filling was not enforced to avoid discouraging them.

## Discussion

This study compared two participatory methods developed to select relevant respiratory pig health indicators while making efficient use of expert’s time. Using the selection of pig respiratory health as a methodological proof of concept, results showed that Method B generated more indicators and provided a more efficient and manageable process than Method A, despite requiring a greater time investment from experts.

### Implementation of the participatory methods

#### Group dynamic.

Results in both methods suggest that the use of small panels composed of colleagues may have influenced group dynamics and the functioning of the participatory process. The interactions between experts facilitated the exchange of perspectives and the explicit formulation of practical knowledge and reasoning that may not have emerged from individual assessments alone. Familiarity can contribute to a supportive discussion environment, facilitating open discussion, disagreements, and the sharing of ideas [26–29]. Similarly, small group sizes can provide participants with greater opportunities to express and explain their views and allow facilitators to explore the reasoning underlying individual assessments in greater depth [30–32]. These characteristics likely facilitated consensus between both panels. Furthermore, involving veterinarians as both animal health experts and future end-users strengthened the validity and overall acceptance of the selected indicators and process [33,34].

Nevertheless, the possibility of a “group think” effect cannot be excluded. Within groups, participants may consciously or unconsciously self-censor or adapt their opinions to those expressed by others in order to preserve group cohesion [35,36]. Such dynamics may facilitate agreement without necessarily reflecting fully independent judgement. Although experts expressed different views and discussed disagreements during the review tasks, the influence of existing professional relationships and group dynamics cannot be excluded [37,38]. For this reason, the term agreement was preferred rather than consensus throughout this study, as the process did not include formal procedures to establish consensus. More structured participatory approaches, such as the Delphi technique, seek to limit some forms of social influence through individual responses, iterative rounds, and controlled or anonymised feedback [36,39]. These features can reduce the influence of dominant participants and conformity pressures but may also limit the direct interactions through which experts explain, challenge, and refine each other’s reasoning. The methods developed in this study therefore represent a trade-off between preserving independent expert judgement and allowing direct exchanges of contextual and practical knowledge.

#### Expert understanding.

Maintaining expert motivation and ensuring a clear understanding of the participatory process were important considerations in the implementation of both methods. Previous studies have similarly highlighted the importance of sustained participant motivation and a clear understanding of the tasks were for effective participation [32,40,41]. The dedicated training provided before the scoring task probably contributed to this objective by clarifying expectations and reducing uncertainties. However, in both methods, the numerous questions and requests for clarification raised by participants indicated that some aspects of the documentation and initial training could be improved. In particular, providing additional context on the overall project and including more examples illustrating how to score indicators could facilitate participants’ understanding of the task.

Likewise, regular interactions with the participant during the approach may also be important. In both methods, facilitators remained available to answer questions and, when applicable, followed expert’s progress during the scoring task. Such interactions may help address uncertainties and limit participant disengagement as previously recommended in the literature [24,42,43]. In future participatory approaches, these interactions will be even more frequent.

#### Time management.

Time management emerged as a key consideration in the implementation of the participatory methods. Veterinarians have limited availability, making the balance between the time required from experts and the quality of their contribution an important challenge when designing participatory approaches.

Results from Method B suggest that distributing the workload over a longer period may offer advantages despite requiring a greater cumulative time commitment from experts. Indeed, a longer scoping session provided more opportunities to clarify the approach and address expert’s questions. The asynchronous scoring task, although it required a greater cumulative time commitment from experts, provided them time to reflect on their assessments, revise their scores and provide detailed rationales. In parallel, the interval between the scoring and review tasks allowed facilitators to conduct descriptive analyses and identify indicators requiring further discussion. Although shorter sessions could reduce the time commitment required from experts, this may come to the detriment to the quality of the information collected [32].

In contrast, Method A illustrated some of the limitations associated with completing all stages of the participatory process within a single session. Time constraints was detrimental to the training, the scoring task or the discussions and overall, the completeness of the final set of relevant respiratory health indicator. Moreover, experts had to carry out a difficult task in a reduced amount of time, which could have limited opportunities for them to reflect on their assessments. As highlighted in the literature, excessive time pressure can encourage experts to seek rapid agreement without fully expressing and exploring contradictory views [44].

Overall, these findings suggest that providing sufficient time for training and scoring indicators may support richer assessments and contributions.

#### Flexibility.

The level of flexibility in both methods played an important role in shaping the outcomes. Although the Method B required a greater cumulative time commitment, experts particularly valued the four-week scoring period, which allowed them to organize the task according to their professional availability. Thus, the burden associated with participation may depend not only on the total time required but also on how flexibly this time can be allocated. This is consistent with recommendations from the literature to limit the burden placed on experts, as excessive work may reduce motivation, and the quality of their contributions [32]. Carrying out the scoring task individually may also have provided an additional advantage by limiting some of the social influences associated with group work, such as the dominance of particular participants or conformity pressures [34,45]. However, flexibility and autonomy need to be balanced with sufficient guidance throughout the process. Previous studies suggest that participant autonomy can promote ownership of the process, whereas appropriate facilitator guidance can help maintain engagement, clarify expectations, and keep participants focused on the objectives of the task [31,46,47]. Method B combined these two dimensions by allowing experts flexibility during the individual scoring task while maintaining contact with the facilitators. This balance between autonomy and guidance may therefore be an important consideration when designing participatory approaches involving professionals with limited availability.

Finally, the use of online meetings in Method B provided additional logistical flexibility. Experts could participate without travelling and could more easily integrate the sessions into their professional schedules, potentially reducing barriers to participation [48]. Although online meetings can sometimes hinder in-depth discussions, it is believed that the effect was limited in this study, as experts knew each other, thus facilitating exchanges [49]. Face-to-face meetings (Method A) remain valuable to foster richer interactions and immediate feedback, but can provoke counterproductive behaviours and be more constraining in terms of time and logistics for veterinarians [36]. The choice between online and face-to-face formats should therefore consider both the opportunities for interaction and the practical constraints associated with expert participation.

### Selection of respiratory health indicators

Combining quantitative summaries of individual assessments with collective discussion (Method B) appeared particularly useful for the selection and characterization of respiratory health indicators. Descriptive statistics (*e.g.,* mean score, percentage of use) and visual representations of score variability (*e.g.,* colour code) provided a structured basis for identifying indicators requiring further discussion. Collective discussions then complemented these quantitative results by eliciting expert rationales, contextual considerations, and practical constraints [34,50,51]. This combination supported not only the selection of indicators but also to their adaptation to practical application. Providing explicit rationales may also strengthen the transparency and interpretability of the selection process [32,36]. Although the review task in Method A also provided additional information on the selected indicators, the absence of descriptive summaries and visual representations of score variability offered less guidance for prioritizing indicators for discussion.

Method B resulted in a larger number of indicators than Method A, providing broader coverage of respiratory health. Indicators independently proposed by experts in Method A were also present in the literature-based list used in Method B. Only three indicators were not present in the initial list used in Method B. They had been either included in the pre-defined list under a different name (*i.e.*, “batch heterogeneity” being similar to “growth retardation”) or kept for a future participatory approach (*i.e.*, “abortion” and “infertility” for the selection of pig reproductive health indicators). Therefore, asking experts to propose themselves indicators did not substantially bring new knowledge in comparison to the initial pre-defined list. However, the number of indicators identified through Method A might be the consequence of delays and methodological constraints rather than a parsimonious or more efficient selection. Time pressure and counterproductive behaviors (*i.e.*, chatter between experts) may have led experts to prioritize familiar or highly salient indicators while excluding less consensual indicators, suggesting a confirmation bias [52]. This could also explain why all the proposed indicators were unanimously considered relevant. Thus, with more time for each step or a better time management, it is believed that experts could have proposed and selected more indicators.

While using a literature-based list of indicators was a strength, allowing experts to complete it with additional indicators proved to be relevant, reduced the likelihood of omissions and capture context-specific and practice-based knowledge that may not be fully represented in the scientific literature. In method B, experts proposed seven additional indicators, demonstrating that a pre-defined list does not necessarily restrict them. Some additions refined or provided alternative formulations of existing indicators (*i.e.*, “cool-seeking behavior” and “hyperthermia”, “wasting away” and “lethargy”), whereas others introduced more specific measures based on clinical practice (*i.e.*, “nasal septum deviation”, “pulmonary oedema”, “tracheitis” and “snout histology”). For instance, snout histology is an uncommon analysis performed if porcine cytomegalovirus is suspected [23]. Finally, “total condemnation” was identified during the narrative review but not included in the scoring matrices, as it was considered not relevant for respiratory health [23]. Allowing experts to add indicators therefore provides an opportunity to challenge, refine, and complement the initial list with practice-based knowledge. The combination of a literature-based list and expert knowledge further optimized the final set of indicators, ensuring an even more relevant and comprehensive selection. Moreover, it likely mitigated anchoring bias, by preventing experts from focusing exclusively on the initial list [53].

The final sets of indicators also illustrate the multidimensional nature of veterinarians’ assessment of respiratory health. Experts selected indicators measured at both the individual and herd levels and included both animal- and resource-based measures. Considering complementary sources of information may contribute to a more comprehensive assessment of animal health [54]. Moreover, in both methods, the importance attributed to post-mortem findings (at the slaughterhouse or during a necropsy) is consistent with the literature, where these measures are increasingly seen as an efficient tool for livestock health surveillance [55].

In Method B, experts also merged several closely related indicators into broader measures considered more applicable under field conditions. Such adaptations illustrate the contribution of expert knowledge in translating indicators identified from the literature into measures applicable to field conditions. However, whether these simplifications improve the feasibility or reliability of the resulting measures will require further evaluation.

Finally, ten indicators initially considered in the assessment of respiratory health were reclassified by experts as systemic health indicators. This reclassification illustrates the overlap between respiratory-specific and general health information and suggests that respiratory health assessment may need to be interpreted alongside indicators reflecting the overall health status of the animals.

## Conclusions

The aim of this study was to identify which participatory method performs well for establishing a comprehensive, agreement-based set of respiratory health indicators under strict time constraints. This study demonstrate that Method B is more suitable than Method A for reaching this objective. Indeed, even though both methods achieved the prerequisite of explicit expert agreement on indicators’ relevance, Method B proved superior by offering a larger and better-characterised final set of respiratory health indicators, while minimizing possible omissions or bias. Combining an evidence-based starting point with an iterative and guided participatory approach revealed to be a strength. Method B not only corroborated the findings of Method A but also highlighted the added value of the narrative review and the possibility to add indicators in providing a better foundation for the selection process. Moreover, beyond its methodological value, this method directly benefits the veterinarians by providing exchanges, allowing experts to share knowledge and identify new, relevant indicators to optimize their on-farm health monitoring. After this work on respiratory health, participatory approaches using the Method B will be conducted to identify and select pig health indicators that are relevant to monitor other biological systems (*i.e.,* digestive, locomotor, urinary, nervous, reproductive or cutaneous health). This work constitutes the first step toward the development of a protocol aggregating health indicators for monitoring pig health in farms in a context of reduced AMU.

## Supporting information

S1 File PDF Documentation sent to the experts prior to the scoping session. Experts were sent a first document presenting the participatory approach, a second one introducing the scoring matrices and scoring instructions, and a third introducing the four characteristics to score and their associated statements.
(PDF)

S2 File Detailed results of the scoring task of pig respiratory health indicators in Method B. The percentage of use (among the number of experts) is indicated for each physiological stage, as well as the mean scores of feasibility, reliability, specificity and sensitivity obtained for each indicator. To facilitate expert interpretation, a color code was applied to distinguish four categories of indicators: (i) low-score indicators (mean ≤ 2), (ii) mid-score indicators (2 < mean < 2.5), (iii) high-score indicators with low variability (mean ≥ 2.5 and SD ≤ 1), and (iv) high-score indicators with high variability (mean ≥ 2.5 and SD > 1).
(PDF)
