# Supplementary material for: Comparative assessment of two participatory approach methods for selecting pig health indicators: A proof-of-concept on respiratory health
Source: PLoS One. 2026 Sep 17;21(9):e0358467. doi: 10.1371/journal.pone.0358467 (PMC13585232; doi:10.1371/journal.pone.0358467)
Supplement: Supplementary file 1 [file pone.0358467.s001.pdf]

Participatory approach

Respiratory health indicators in pigs

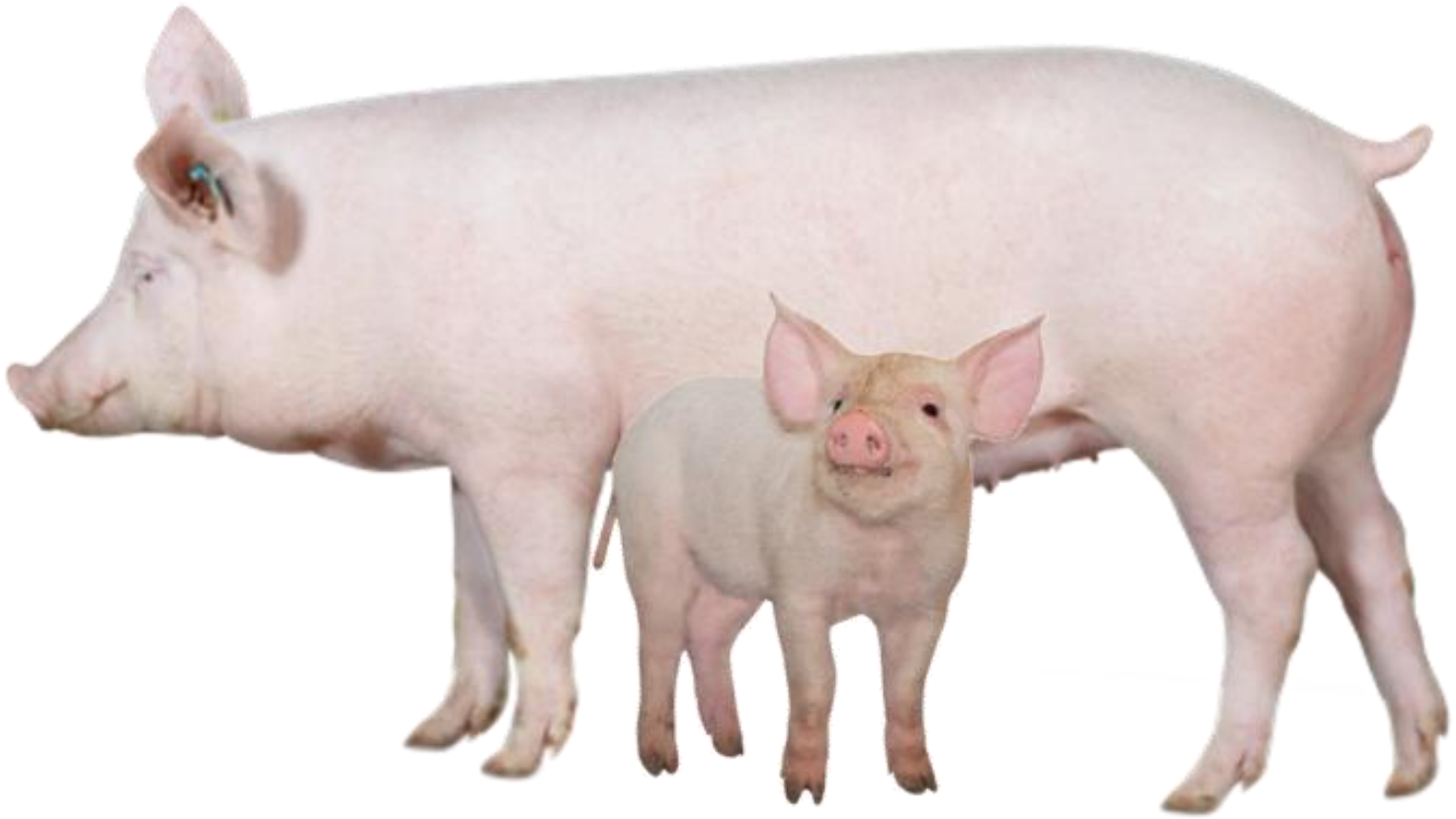

Théo DESSIER, Emélie SUAZO, Catherine BELLOC, Mily LEBLANC-MARIDOR, Nikky Millar

BIOEPAR INRAE Oniris

Context

Virtuous use of antibiotics without jeopardizing animal health and welfare

ROADMAP project --> Living Lab to address this issue

Development of a farm-level assessment for animal health, welfare and antibiotic use. With it, veterinarians and farmers can manage antibiotic use effectively.

Identifying the most relevant indicators

We want to create a list of indicators used by veterinarians and identify the most relevant ones based on their characteristics:

Specificity

Sensitivity

Reliability

Feasibility

This is the aim of this participatory approach

Indicators

↓

Assessment protocol
(health, welfare and antibiotic use)

↓

Virtuous use of antibiotics, with animal welfare and health preserved

Glossary

	Definition	Statements
**Feasibility**	Refers to the practicality of the indicator in terms of time, equipment, operators' skills, and costs.	**All categories**: The resources required to measure the indicator are reasonable.
**Reliability**	Refers to the capacity of the indicator to provide consistent results, regardless of the operator, the equipment used, or the time of measurement.	**All categories**: The measurement of this indicator is repeatable and reproductible.
**Specificity**	Refers to the capacity of the indicator to measure what it is intended to measure, and not something else.	**Clinical signs/Necropsy data/Slaughterhouse data:** This indicator can (almost) only be observed during this affection.
		**Biological analysis:** This indicator (i.e. analysis) gives few false positives.
		**Performance data:** This performance data is (almost) only impacted by the concerned affection.
		**Environmental data:** This environmental data is closely related to the affection.
**Sensitivity**	Refers to the ability of the indicator to detect variations in health status, even when these variations are small.	**Clinical signs/Necropsy data/Slaughterhouse data:** This indicator can be observed for (almost) all cases of this affection.
		**Biological analysis:** This analysis gives few false negatives.
		**Performance data:** This performance data is impacted for (almost) all cases of this affection.
		**Environmental data:** This environmental data influences (almost) every time the onset of this affection.

Participatory approach's schedule

Before	Scoping session	Scoring task	Review task
Sending documents	Introduction to the study and its context		Presentation and discussion of the results
	Scoring instructions	Assessment of indicators' relevance through scoring matrices (individual work)	Selection of the most relevant indicators
	Questions & Answers		Feedback on the approach
	**1 hour (online session)**	**A few weeks**	**2 hours (online session)**

Indicators' assessment

For this participatory approach, we want to determine which indicators are relevant to assess respiratory diseases in pigs.

We will discuss the following diseases: enzootic pneumonia, pleuropneumonia, influenza, PRRS, Glässer's disease and atrophic rhinitis.

How does the scoring work?
Using a scoring matrix, we will ask you, for each indicator, whether you use it, how you use it … but above all, you will assess that indicator through four characteristics: sensitivity, specificity, reliability and feasibility.

Indicators are classified into six categories. There will therefore be six matrices to complete:
"Clinical signs"
"Necropsy data"
"Biological analysis"
"Performance data"
"Slaughterhouse data"
"Environmental data"

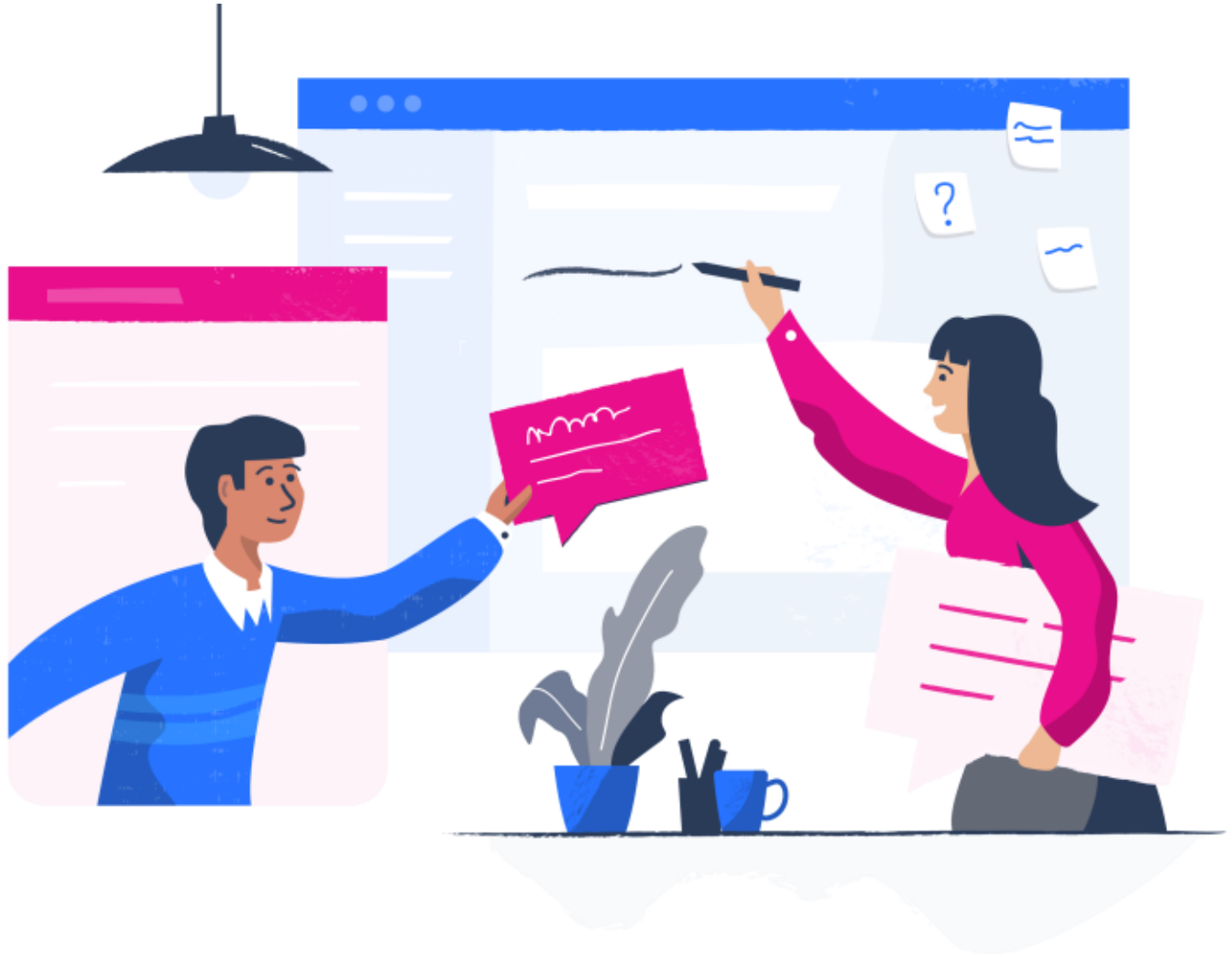

Thank you for your participation !

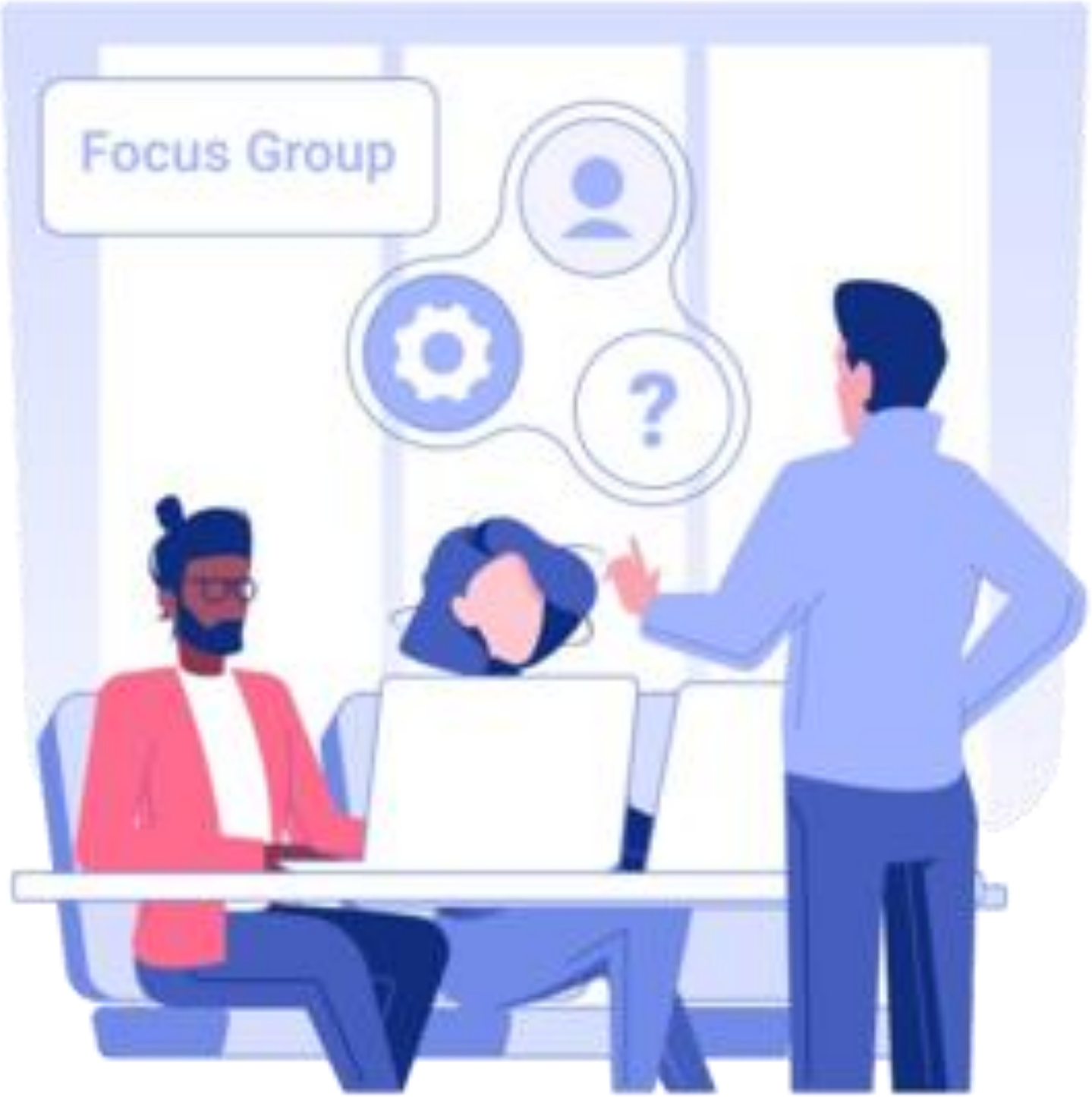

Here is a scoring matrix that you'll have to complete during the scoring task (below, an extract from the "clinical signs" matrix with examples). There will be a total of six matrices to complete (one for each category of indicator). The six matrices are identical.

The list of indicators to assess. They are divided into six tables: clinical signs, biological data, necropsy data, slaughterhouse data, performance data and environmental data.

Do you use this indicator to study respiratory disorders in pigs?

Any comment about the indicator or your scores?

Indicator		Use ?	If not, why ?	If yes, how ?	Physiological stage				Feasibility (The resources required to measure the indicator are reasonable.)	Reliability (The measurement of this indicator is repeatable and reproductible.)	Specificity (This indicator can (almost) only be observed during this affection.)						Sensitivity (This indicator can be observed for (almost) all cases of this affection.)						Comments ?
					Suckling piglets	Weaners	Finishers	Sows			Enzootic pneumonia	Pleuropneumonia	Influenza	PRRS	Glässer disease	Atrophic rhinitis	Enzootic pneumonia	Pleuropneumonia	Influenza	PRRS	Glässer disease	Atrophic rhinitis	
Respiratory clinical signs	Coughing	X		Dozen of pigs, quantitative measure (number of occurrence per minute)	X		X	X	2	2	4	3	2	1	1	2	1	2	3	3	3	1	For sows, only relevant in case of influenza
	Sneezing		Not relevant																				
	Nasal discharge	X		All the pens, quantitative measure (total proportion of pigs)	X	X			3	4	4	3	2	2	1	4	4	3	2	2	1	1	
Other indicators																							

You can add and assess indicators that are not cited but important for pig respiratory health.

For which production stage is this indicator relevant to measure?
Suckling piglets
Growing pigs
Finishers
Sows

The relevance of the indicator must be assessed according to four statements related to four characteristics: feasibility, reliability, specificity and sensitivity. Scoring is on a scale of 1 to 4. For feasibility and accuracy, the indicator must be assessed in the context of general pig respiratory health. For specificity and sensitivity, the indicator must be assessed in the context of respiratory diseases.

Enzootic pneumonia / Pleuropneumonia / Influenza / Porcine reproductive and respiratory syndrome (PRRS) / Glässer disease /Atrophic rhinitis

How to score (below, an extract of the "clinical signs" matrix with false scorings). The scoring method will be the same for all matrices.

Indicators are scored on a scale of 1 to 4. For each indicator, statements are listed in the header alongside their corresponding characteristics (feasibility, accuracy, specificity, sensitivity). You will need to indicate your level of agreement with each statement in relation to the indicator you are assessing.

For example, for the "clinical signs" matrix, feasibility is assessed using the statement "The resources required to measure the indicator are reasonable". Thus, if you are assessing the feasibility of "nasal discharge" in the context of atrophic rhinitis and you totally agree with this statement, you should give a score of 4.

Indicator		Use ?	If not, why ?	If yes, how ?	Physiological stage				Feasibility (*The resources required to measure the indicator are reasonable.*)	Reliability (*The measurement of this indicator is repeatable and reproductible.*)	Specificity (*This indicator can (almost) only be observed during this affection.*)						Sensitivity (*This indicator can be observed for (almost) all cases of this affection.*)						Comments ?
					Suckling piglets	Weaners	Finishers	Sows			Enzootic pneumonia	Pleuropneumonia	Influenza	PRRS	Glässer disease	Atrophic rhinitis	Enzootic pneumonia	Pleuropneumonia	Influenza	PRRS	Glässer disease	Atrophic rhinitis	
Respiratory clinical signs	Coughing	X		Dozen of pigs, quantitative measure (number of occurrence per minute)	X		X	X	2	2	4	3	2	1	1	2	1	2	3	3	3	1	For sows, only relevant in case of influenza
	Sneezing		Not relevant																				
	Nasal discharge	X		All the pens, quantitative measure (total proportion of pigs)	X	X			3	4	4	3	2	2	1	4	4	3	2	2	1	1	
Other indicators																							

Reminder of the scoring scale

1) ***Total disagreement***
2) ***Partial disagreement***
3) ***Partial agreement***
4) ***Total agreement***

Definitions of the characteristics and statements for each category

	Definition	Statements
**Feasibility**	Refers to the practicality of the indicator in terms of time, equipment, operators' skills, and costs.	**All categories**: The resources required to measure the indicator are reasonable.
**Reliability**	Refers to the capacity of the indicator to provide consistent results, regardless of the operator, the equipment used, or the time of measurement.	**All categories**: The measurement of this indicator is repeatable and reproductible.
**Specificity**	Refers to the capacity of the indicator to measure what is intended to be measured, and not something else.	**Clinical signs/Necropsy data/Slaughterhouse data:** This indicator can (almost) only be observed during this affection.
		**Biological analysis:** This indicator (i.e. analysis) gives few false positives.
		**Performance data:** This performance data is (almost) only impacted by the concerned affection.
		**Environmental data:** This environmental data is closely related to the affection.
**Sensitivity**	Refers to the ability of the indicator to detect variations in health status, even when these variations are small.	**Clinical signs/Necropsy data/Slaughterhouse data:** This indicator can be observed for (almost) all cases of this affection.
		**Biological analysis:** This analysis gives few false negatives.
		**Performance data:** This performance data is impacted for (almost) all cases of this affection.
		**Environmental data:** This environmental data influences (almost) every time the onset of this affection.
