# Supplementary material for: Comparative assessment of two participatory approach methods for selecting pig health indicators: A proof-of-concept on respiratory health
Source: PLoS One. 2026 Sep 17;21(9):e0358467. doi: 10.1371/journal.pone.0358467 (PMC13585232; doi:10.1371/journal.pone.0358467)
Supplement: Supplementary file 2 [file pone.0358467.s002.pdf]

| x̄>= 2.5 with SD <= 1 | x̄ >= 2.5 with SD > 1 | 2 < x̄ < 2.5 |
|---|---|---|

**Clinical signs**

| Indicators | Physiological stage | | | | Feasibility | Reliability | Enzootic pneumonia | | APP infection | | Influenza | | PRRS | | Glässer Disease | | Atrophic rhinitis | |
|---|---|---|---|---|---|---|---|---|---|---|---|---|---|---|---|---|---|---|
| | Sow | Growing pig | Finisher | Sow | | | Specificity | Sensitivity | Specificity | Sensitivity | Specificity | Sensitivity | Specificity | Sensitivity | Specificity | Sensitivity | Specificity | Sensitivity |
| **Assessed by five participants** | | | | | **Assessed by five participants** | | | | | | | | | | | | | |
| Sneezing | 60 | 100 | 100 | 80 | 4,00 | 3 | 1,40 | 1,40 | 1 | 1 | 3 | 3,20 | 1,40 | 1,60 | 2 | 2 | 3 | 3,40 |
| Coughing | 100 | 100 | 100 | 100 | 4 | 3 | 3,40 | 3,40 | 1,40 | 1,20 | 2,60 | 2,40 | 2,40 | 2 | 2,20 | 2 | 1 | 1 |
| Dyspnea | 60 | 100 | 100 | 80 | 4 | 3,75 | 3 | 2,25 | 3,67 | 3 | 3,25 | 2,75 | 2,25 | 1,75 | 3,25 | 3 | 1,50 | 1 |
| Polypnea | 40 | 80 | 80 | 80 | 4 | 3,75 | 2,67 | 2,33 | 2,67 | 3,00 | 3,67 | 3,00 | 2,33 | 2 | 2,33 | 3,00 | 1,33 | 1 |
| Nasal discharge | 100 | 100 | 100 | 100 | 3,60 | 3,20 | 1,60 | 1,60 | 1,40 | 1,60 | 3,20 | 3,20 | 2 | 1,80 | 2,40 | 2,20 | 2,80 | 2,60 |
| Epistaxis | 20 | 80 | 100 | 60 | 4 | 3,60 | 1 | 1 | 2,40 | 1,80 | 1 | 1 | 1 | 1 | 1 | 1 | 3,40 | 2,40 |
| Twisted snout | 20 | 80 | 100 | 40 | 3,40 | 2,80 | 1 | 1 | 1 | 1 | 1 | 1 | 1 | 1 | 1 | 1 | 4 | 2 |
| Feeding difficulties | 20 | 100 | 100 | 100 | 3,50 | 3 | 2 | 1,25 | 3,50 | 3,50 | 3,40 | 3,40 | 2,00 | 1,60 | 2,40 | 2,00 | 1,25 | 1 |
| Lethargy | 80 | 100 | 100 | 100 | 3,60 | 2,80 | 1,50 | 1,25 | 3,75 | 3,25 | 3,40 | 3,20 | 2,20 | 1,80 | 2,80 | 2,20 | 1,25 | 1 |
| Hyperthermia | 40 | 80 | 80 | 100 | 3,50 | 3,25 | 1,67 | 1 | 3,67 | 3,33 | 3,75 | 3,75 | 3 | 2 | 3,25 | 2,75 | 1 | 1 |
| Growth retardation | 60 | 100 | 100 | 0 | 3,60 | 2,80 | 2,60 | 2,40 | 1,50 | 2,00 | 2 | 1,60 | 2,60 | 2 | 2 | 2,40 | 2,60 | 2,80 |
| Rough hair coat | 80 | 100 | 100 | 40 | 3,80 | 3,50 | 2,33 | 2 | 1,33 | 1 | 2 | 1,67 | 3,00 | 2 | 2,33 | 2,33 | 3,00 | 2,33 |
| Mortality | 80 | 100 | 100 | 80 | 3,75 | 3,50 | 1,25 | 1 | 3,50 | 2,75 | 1,75 | 1,25 | 2,50 | 1,75 | 3,25 | 2,25 | 1 | 1 |
| **Assessed by four participants** | | | | | **Assessed by four participants** | | | | | | | | | | | | | |
| Conjunctivitis | 50 | 75 | 75 | 75 | 3,75 | 3,25 | 1,75 | 1 | 1 | 1 | 2 | 1,50 | 2,25 | 1,50 | 2 | 1,50 | 2,75 | 2 |
| Cyanosis | 75 | 100 | 100 | 100 | 4 | 3,75 | 1 | 1 | 3,25 | 1,75 | 1,75 | 1,25 | 2,50 | 1,50 | 2 | 1,25 | 1 | 1 |
| Huddling | 100 | 100 | 75 | 0 | 3,50 | 2,50 | 1,75 | 1,75 | 1 | 1 | 2,25 | 1,50 | 1,75 | 1,25 | 2,25 | 2 | 1 | 1 |
| Abnormal vocalizations | 50 | 100 | 75 | 75 | 3,25 | 2,75 | 1,00 | 1,00 | 1,50 | 1 | 1,25 | 1 | 1 | 1 | 2 | 1,25 | 2 | 1,25 |
| **Assessed by three participants** | | | | | **Assessed by three participants** | | | | | | | | | | | | | |
| Anemia | 33 | 67 | 67 | 67 | 3 | 2,50 | 1,33 | 1,33 | 2,67 | 2 | 2 | 1,33 | 2,33 | 1,67 | 2,67 | 2 | 1,67 | 1 |
| Panting | 33 | 100 | 100 | 67 | 4 | 2,67 | 1,67 | 1,33 | 2,00 | 1,67 | 3,33 | 2 | 1,67 | 1,33 | 2,67 | 2 | 1,33 | 1 |
| Orthopnea | 33 | 100 | 100 | 100 | 4 | 3,67 | 1 | 1,5 | 3,50 | 3,50 | 2 | 3 | 1,50 | 2 | 1,50 | 2 | 1 | 1 |
| Pain posture | 33 | 67 | 100 | 100 | 4 | 3,67 | 2,50 | 1 | 3 | 1,67 | 2 | 1 | 1,50 | 1 | 2,67 | 1 | 1 | 1 |
| **Assessed by two participants** | | | | | **Assessed by two participants** | | | | | | | | | | | | | |
| Wasting away | 50 | 100 | 100 | 50 | 4 | 4 | 1 | 1 | 1 | 1 | 2 | 1 | 2 | 1 | 1 | 1 | 2 | 1 |
| Dehydration | 100 | 100 | 100 | 0 | 3,50 | 2,50 | 2 | 1 | 1 | 1 | 1,50 | 1 | 1 | 1 | 2 | 1 | 1 | 1 |
| Tachypnae | 50 | 100 | 100 | 100 | 2,50 | 2 | 2 | 2 | 2,50 | 2,50 | 2,50 | 2,50 | 1 | 1 | 2,50 | 2,50 | 1 | 1 |
| **Assessed by one participant** | | | | | **Assessed by one participant** | | | | | | | | | | | | | |
| Bradypnea | 100 | 100 | 100 | 100 | 4 | 3 | 3 | 1 | 4 | 1 | 1 | 1 | 1 | 1 | 4 | 1 | 1 | 1 |
| Hypothermia | 100 | 100 | 100 | 100 | 4 | 3 | 2 | 1 | 4 | 1 | 1 | 1 | 1 | 1 | 4 | 1 | 1 | 1 |
| Cool-seeking behaviour | 0 | 100 | 100 | 0 | 3 | 3 | 1 | 1 | 2 | 3 | 3 | 3 | 2 | 1 | 3 | 1 | 1 | 1 |
| Shivering | 100 | 100 | 100 | 100 | 4 | 3 | 1 | 1 | 2 | 1 | 3 | 1 | 1 | 1 | 4 | 1 | 1 | 1 |
| **Not assessed** | | | | | **Not assessed** | | | | | | | | | | | | | |
| Bradycardia | | | | | | | | | | | | | | | | | | |
| Tachycardia | | | | | | | | | | | | | | | | | | |

| $\bar{x}$ >= 2.5 with SD <= 1 | $\bar{x}$ >= 2.5 with SD > 1 | 2 < $\bar{x}$ < 2.5 |
|---|---|---|

| Biological analysis | | | | | | | | | | | | | | |
|---|---|---|---|---|---|---|---|---|---|---|---|---|---|---|
| **Indicators** | **Feasibility** | **Reliability** | **Enzootic pneumonia** | | **APP infection** | | **Influenza** | | **PRRS** | | **Glässer Disease** | | **Atrophic rhinitis** | |
| | | | **Specificity** | **Sensitivity** | **Specificity** | **Sensitivity** | **Specificity** | **Sensitivity** | **Specificity** | **Sensitivity** | **Specificity** | **Sensitivity** | **Specificity** | **Sensitivity** |
| Bacterial culture | 3,2 | 3 | 4 | 2,5 | 4 | 3,8 | | | | | 3,8 | 2,2 | 3,5 | 3 |
| Bacterial identification | 3,5 | 3,25 | | | 3,33 | 3,33 | | | | | 3,67 | 2 | | |
| Mass spectrometry | 3,75 | 3,5 | | | 4 | 4 | | | | | 4 | 3 | 4 | 3 |
| PCR | 3,6 | 2,8 | 3,4 | 2,8 | 3,5 | 3,5 | 4 | 2,4 | 3,8 | 3,75 | 3,5 | 2 | 3,75 | 2,5 |
| Genotyping | 2,75 | 3 | | | | | 4 | 4 | 4 | 4 | 4 | 2,5 | | |
| Viral isolation | 2 | 3 | | | | | 4 | 4 | | | | | | |
| Immunofluorescence | 3 | 2,75 | 4 | 4 | | | | | | | | | | |
| Indirect ELISA | 3 | 3,25 | | | | | | | | | 2 | 2 | 4 | 2 |
| Direct ELISA | 3,75 | 3,25 | 3,33 | 3 | 3 | 4 | 3 | 2 | 3 | 3,75 | 2,5 | 2,5 | | |
| Hemagglutination inhibition | 4 | 3,33 | | | | | 3 | 3,5 | | | | | | |
| Virus neutralization assay | 2 | 3,33 | | | | | 4 | 4 | | | | | | |
| Lung histology | 3,6 | 3,2 | 3,2 | 2,8 | 3,25 | 3 | 2,75 | 2,5 | 2,5 | 2,25 | 2,67 | 1,33 | | |
| Lymphatic node histology | 3,75 | 3,25 | 3,33 | 3 | 4 | 4 | 3 | 3 | 3 | 3 | 1 | 1 | | |
| Nasal cavity histology | 4 | 3 | | | | | | | | | | | 4 | 2 |
| **Not assessed** | **Not assessed** | | | | | | | | | | | | | |
| *In situ* hybridization | | | | | | | | | | | | | | |
| Immunohistochemistry | | | | | | | | | | | | | | |
| Parasitological analysis | | | | | | | | | | | | | | |
| Complement fixation | | | | | | | | | | | | | | |
| Complete blood count | | | | | | | | | | | | | | |
| Measure of anemia | | | | | | | | | | | | | | |
| Cell study | | | | | | | | | | | | | | |
| Measure of blood parameters | | | | | | | | | | | | | | |
| Measure of blood oxygen | | | | | | | | | | | | | | |

x̄ >= 2.5 with SD <= 1 | x̄ >= 2.5 with SD > 1 | 2 < x̄ < 2.5

**Necropsy data**

| Indicators | Physiological stage: Sow | Physiological stage: Growing pig | Physiological stage: Finisher | Physiological stage: Sow | Feasibility | Reliability | Enzootic pneumonia: Specificity | Enzootic pneumonia: Sensitivity | APP infection: Specificity | APP infection: Sensitivity | Influenza: Specificity | Influenza: Sensitivity | PRRS: Specificity | PRRS: Sensitivity | Glässer Disease: Specificity | Glässer Disease: Sensitivity | Atrophic rhinitis: Specificity | Atrophic rhinitis: Sensitivity |
|---|---|---|---|---|---|---|---|---|---|---|---|---|---|---|---|---|---|---|
| **Assessed by five participants** | | | | | | | | | | | | | | | | | | |
| Pleuritis | 40 | 80 | 100 | 60 | 4 | 3 | 1,33 | 1,33 | 3,5 | 4 | 1,25 | 1,25 | 1 | 1,5 | 3,25 | 3,25 | 1 | 1 |
| Pneumonia | 60 | 100 | 100 | 100 | 4 | 3 | 3 | 3,6 | 2,2 | 2,2 | 2,2 | 3,2 | 2,2 | 2,6 | 2 | 2,2 | 1 | 1 |
| Lymphatic node lesion | 40 | 100 | 100 | 80 | 3,8 | 3 | 1,6 | 2,2 | 1,8 | 2,8 | 1,4 | 2,2 | 1,4 | 2,6 | 1,8 | 3 | 1,2 | 1,4 |
| Cardiac lesion | 40 | 100 | 100 | 60 | 4 | 3,6 | 1 | 1 | 2 | 1,25 | 1 | 1 | 1 | 1,2 | 2,8 | 2,4 | 1 | 1 |
| Hepatic lesion | 40 | 100 | 100 | 80 | 3,8 | 3,2 | 1 | 1 | 1,25 | 1 | 1 | 1 | 1 | 1,25 | 2,2 | 1,8 | 1 | 1 |
| **Assessed by four participants** | | | | | | | | | | | | | | | | | | |
| Pulmonary abscess | 50 | 100 | 100 | 75 | 4 | 3,25 | 1 | 1 | 2,33 | 2,67 | 1 | 1 | 1 | 1 | 1 | 1 | 1 | 1 |
| Atrophy nasal turbinates | 50 | 75 | 100 | 25 | 2,25 | 3 | 1 | 1 | 1 | 1 | 1 | 1 | 1 | 1 | 1 | 1 | 3,75 | 3 |
| **Assessed by three participants** | | | | | | | | | | | | | | | | | | |
| Rhinitis | 33 | 67 | 100 | 33 | 2,33 | 2,67 | 1 | 1 | 1 | 1 | 1 | 1 | 1 | 1 | 1 | 1 | 3,67 | 3 |
| **Assessed by one participant** | | | | | | | | | | | | | | | | | | |
| Pulmonary oedema | 0 | 100 | 100 | 100 | 4 | 4 | | | | | 1 | 2 | 1 | 2 | | | | |
| Tracheitis | 0 | 0 | 100 | 0 | 3 | 2 | 1 | 1 | 1 | 1 | 3 | 1 | 1 | 1 | 1 | 1 | 1 | 1 |

| $\bar{x}$ >= 2.5 with SD <= 1 | $\bar{x}$ >= 2.5 with SD > 1 | 2 < $\bar{x}$ < 2.5 |
|---|---|---|

| | Indicators | Physiological stage | | | | Feasibility | Reliability | Enzootic pneumonia | | APP infection | | Influenza | | PRRS | | Glässer Disease | | Atrophic rhinitis | |
|---|---|---|---|---|---|---|---|---|---|---|---|---|---|---|---|---|---|---|---|
| | | Sow | Growing pig | Finisher | Sow | | | Specificity | Sensitivity | Specificity | Sensitivity | Specificity | Sensitivity | Specificity | Sensitivity | Specificity | Sensitivity | Specificity | Sensitivity |
| Slaughterhouse data | **Assessed by five participants** | | | | | **Assessed by five participants** | | | | | | | | | | | | | |
| | Pleuritis | 0 | 0 | 80 | 20 | 2,4 | 3,2 | 1,25 | 1,25 | 3,4 | 3,8 | 1 | 1,75 | 1 | 1 | 2 | 2 | 1 | 1 |
| | Pneumonia | 0 | 0 | 80 | 20 | 2,2 | 3,2 | 3 | 3,4 | 2 | 2,5 | 2,25 | 1,75 | 2,25 | 1,75 | 1,75 | 1,5 | 1 | 1 |
| | **Assessed by four participants** | | | | | **Assessed by four participants** | | | | | | | | | | | | | |
| | Hepatic lesion | 0 | 0 | 75 | 25 | 2,333333 | 2,666667 | 1 | 1 | 1 | 1 | 1 | 1 | 1 | 1 | 1,666667 | 1,333333 | 1 | 1 |
| | **Assessed by three participants** | | | | | **Assessed by three participants** | | | | | | | | | | | | | |
| | Pulmonary abcess | 0 | 0 | 100 | 0 | 2,25 | 3 | 1 | 1 | 2 | 2 | 1 | 1,25 | 1 | 1 | 1 | 1 | 1 | 1 |
| | **Assessed by two participants** | | | | | **Assessed by two participants** | | | | | | | | | | | | | |
| | Rhinitis | 0 | 0 | 100 | 0 | 1,5 | 2,5 | 1 | 1 | 1 | 1 | 1 | 1 | 1 | 1 | 1 | 1 | 2,5 | 1,5 |
| | Atrophy nasal turbinates | 0 | 0 | 100 | 0 | 2 | 3 | 1 | 1 | 1 | 1 | 1 | 1 | 1 | 1 | 1 | 1 | 3,333333 | 2,666667 |
| | Cardiac lesion | 0 | 0 | 100 | 0 | 2,5 | 3 | 1 | 1 | 2,5 | 1,5 | 1 | 1 | 1 | 1 | 2,5 | 1,5 | 1 | 1 |
| | **Assessed by one participant** | | | | | **Assessed by one participant** | | | | | | | | | | | | | |
| | Deviated nasal septum | 0 | 0 | 100 | 0 | 3 | 4 | 1 | 1 | 1 | 1 | 1 | 1 | 1 | 1 | 1 | 1 | 4 | 3 |
| | Lymphatic node lesion | 0 | 0 | 100 | 0 | 3 | 4 | 3 | 4 | 3 | 4 | 3 | 4 | 3 | 4 | 3 | 4 | 2 | 1 |
| | Total condemnations | 0 | 0 | 100 | 0 | 2 | 2 | 1 | 1 | 2 | 1 | 1 | 1 | 1 | 1 | 1 | 1 | 1 | 1 |

| $\bar{x} >= 2.5$ with SD <= 1 | $\bar{x} >= 2.5$ with SD > 1 | $2 < \bar{x} < 2.5$ |
|---|---|---|

| | Indicators | Physiological stage | | | | Feasibility | Reliability | Enzootic pneumonia | | APP infection | | Influenza | | PRRS | | Glässer Disease | | Atrophic rhinitis | |
|---|---|---|---|---|---|---|---|---|---|---|---|---|---|---|---|---|---|---|---|
| | | Sow | Growing pig | Finisher | Sow | | | Specificity | Sensitivity | Specificity | Sensitivity | Specificity | Sensitivity | Specificity | Sensitivity | Specificity | Sensitivity | Specificity | Sensitivity |
| Performance data | **Assessed by five participants** | | | | | **Assessed by five participants** | | | | | | | | | | | | | |
| | Prevalence of morbidity | 60 | 80 | 100 | 100 | 2,8 | 2,8 | 3 | 2,8 | 2,2 | 2,6 | 3,6 | 3,6 | 3,2 | 2,8 | 2,8 | 2,8 | 2,4 | 2,6 |
| | Prevalence of mortality | 60 | 80 | 100 | 100 | 3,6 | 3,6 | 1,2 | 1 | 3,4 | 3,2 | 1,8 | 2 | 2 | 2 | 3 | 3 | 1,2 | 1,2 |
| | Daily weight gain | 20 | 80 | 100 | 20 | 3,2 | 3,8 | 2,8 | 3 | 2 | 2 | 2,8 | 3 | 2,6 | 2,8 | 2,2 | 2,4 | 2,8 | 2,8 |
| | Feed conversion ratio | 0 | 80 | 100 | 20 | 2,8 | 3,6 | 3 | 3 | 1,8 | 2,2 | 2,8 | 2,4 | 2,8 | 2,8 | 2,6 | 2,2 | 3 | 2,8 |
| | Antimicrobial use | 40 | 80 | 80 | 100 | 3 | 3 | 2,6 | 2,8 | 3 | 3,4 | 1,8 | 2 | 1,4 | 1,6 | 3 | 3,2 | 2 | 2 |
| | **Assessed by three participants** | | | | | **Assessed by three participants** | | | | | | | | | | | | | |
| | Weight | 33 | 33 | 100 | 33 | 3 | 3,333333 | 3 | 3 | 1,333333 | 2 | 2,666667 | 3 | 2,333333 | 2,666667 | 2,333333 | 2,333333 | 2,666667 | 2,666667 |

| x̄ >= 2.5 with SD <= 1 | x̄ >= 2.5 with SD > 1 | 2 < x̄ < 2.5 |
|---|---|---|

| | Indicators | Physiological stage | | | | Feasibility | Reliability | Enzootic pneumonia | | APP infection | | Influenza | | PRRS | | Glässer Disease | | Atrophic rhinitis | |
|---|---|---|---|---|---|---|---|---|---|---|---|---|---|---|---|---|---|---|---|
| | | Sow | Growing pig | Finisher | Sow | | | Specificity | Sensitivity | Specificity | Sensitivity | Specificity | Sensitivity | Specificity | Sensitivity | Specificity | Sensitivity | Specificity | Sensitivity |
| **Environmental data** | **Assessed by three participants** | | | | | **Assessed by three participants** | | | | | | | | | | | | | |
| | Density | 33 | 100 | 100 | 33 | 3,7 | 4 | 2,3 | 2,7 | 2,7 | 2,7 | 2,7 | 2,7 | 2,7 | 2,7 | 2,7 | 2,7 | 2,3 | 2,7 |
| | Ambient temperature | 33 | 100 | 100 | 33 | 3,3 | 3,7 | 2,7 | 2,7 | 2,7 | 2,7 | 2,7 | 2,7 | 2,7 | 2,7 | 2,7 | 2,7 | 2,7 | 2,7 |
| | Humidity | 67 | 100 | 100 | 67 | 2,3 | 3,5 | 2,7 | 2,7 | 2,7 | 2,7 | 2,7 | 2,7 | 2,7 | 2,7 | 2,7 | 2,7 | 2,7 | 2,7 |
| | Dust concentration in the air | 67 | 100 | 100 | 67 | 3 | 3 | 2,7 | 2,7 | 2,7 | 2,7 | 2,7 | 2,7 | 2,7 | 2,7 | 2,7 | 2,7 | 2,7 | 2,7 |
| | Gilt quarantine | 67 | 33 | 33 | 100 | 4 | 3,7 | 3 | 2,3 | 2,3 | 2,3 | 3 | 2,3 | 3 | 2,3 | 2,3 | 2,3 | 2,3 | 2,3 |
| | Quarantine of sick animals | 67 | 100 | 100 | 67 | 3,3 | 3,7 | 2,3 | 2,3 | 3 | 2,3 | 2,3 | 2,3 | 2,3 | 2,3 | 3 | 2,3 | 2,3 | 2,3 |
| | Batch mixing | 67 | 100 | 100 | 67 | 3 | 2,3 | 2,7 | 2,7 | 2,7 | 2,7 | 2,7 | 2,7 | 2,7 | 2,7 | 2,7 | 2,7 | 2,7 | 2,7 |
| | Cleaning protocol | 67 | 100 | 100 | 67 | 2,7 | 2 | 2,7 | 2,7 | 3,3 | 2,7 | 2,7 | 2,7 | 2,7 | 2,7 | 3,3 | 2,7 | 2,7 | 2,7 |
| | Room drying | 67 | 100 | 100 | 67 | 2,7 | 2 | 2,7 | 2,7 | 2,7 | 2,7 | 2,7 | 2,7 | 2,7 | 2,7 | 2,7 | 2,7 | 2,7 | 2,7 |
| | Room disinfection | 67 | 100 | 100 | 67 | 2,7 | 2,3 | 2,7 | 2,7 | 3,3 | 2,7 | 2,7 | 2,7 | 2,7 | 2,7 | 3,3 | 2,7 | 2,7 | 2,7 |
| | Hygiene of animals | 67 | 100 | 100 | 33 | 4 | 2 | 2,7 | 2,7 | 2,7 | 2,7 | 2,7 | 2,7 | 2,7 | 2,7 | 2,7 | 2,7 | 2,7 | 2,7 |
| | Hygiene of environment | 67 | 100 | 100 | 67 | 4 | 2 | 2,7 | 2,7 | 2,7 | 2,7 | 2,7 | 2,7 | 2,7 | 2,7 | 2,7 | 2,7 | 2,7 | 2,7 |
| | Vaccination protocol | 67 | 100 | 67 | 100 | 3,7 | 3,3 | 2,7 | 2,7 | 2,7 | 2,7 | 2 | 2 | 2,3 | 2,3 | 2,7 | 2,7 | 3,0 | 3,0 |
| | Health status of animals | 67 | 33 | 33 | 100 | 3 | 3 | 3,3 | 2,7 | 3,3 | 3,0 | 2,7 | 2,7 | 3 | 3 | 3,3 | 3,0 | 2,333333 | 2,3 |
| | Nearby pig farms | 33 | 100 | 100 | 100 | 2,7 | 3,7 | 3,0 | 2,3 | 2 | 2,3 | 3,3 | 2,7 | 3,3 | 2,7 | 2 | 2 | 1 | 1 |
| | Hygiene lock | 67 | 100 | 100 | 100 | 4 | 3,3 | 2 | 2 | 2 | 2,7 | 2,7 | 2,7 | 2,7 | 2,7 | 2 | 2 | 2 | 2 |
| | **Assessed by two participants** | | | | | **Assessed by two participants** | | | | | | | | | | | | | |
| | Clothes types | 100 | 100 | 100 | 100 | 4 | 3 | 2,5 | 2,5 | 2,5 | 3,5 | 3,5 | 3,5 | 3,5 | 3,5 | 2,5 | 2,5 | 2,5 | 2,5 |
| | Ventilation | 100 | 100 | 100 | 50 | 2 | 3,5 | 3,5 | 3,5 | 3,5 | 3,5 | 3,5 | 3,5 | 3,5 | 3,5 | 3,5 | 3,5 | 3,5 | 3,5 |
| | Carbon dioxide concentration in the air | 100 | 100 | 100 | 50 | 2 | 3,5 | 3,5 | 3,5 | 3,5 | 3,5 | 3,5 | 3,5 | 3,5 | 3,5 | 3,5 | 3,5 | 3,5 | 3,5 |
| | Ammoniac concentration in the air | 100 | 100 | 100 | 50 | 2 | 3,5 | 3,5 | 3,5 | 3,5 | 3,5 | 3,5 | 3,5 | 3,5 | 3,5 | 3,5 | 3,5 | 3,5 | 3,5 |
| | Floor type | 50 | 100 | 100 | 50 | 4 | 3 | 3 | 3 | 3 | 3 | 3 | 3 | 3 | 3 | 3 | 3 | 3 | 3 |
